# Removal of Cd(ii) from aqueous solution by ferrate-modified sludge biochar: optimization of preparation conditions, adsorption performance, and mechanism

**DOI:** 10.1039/d6ra00674d

**Published:** 2026-05-06

**Authors:** Tao Long, Xinwei Zuo, Yunping Ji, Changquan Wang

**Affiliations:** a College of Resources, Sichuan Agricultural University Chengdu 611130 China chang10393@163.com; b China Nuclear Industry Survey Design & Research Co., Ltd Zhengzhou 450002 China; c China Railway First Survey and Design Institute Group Co., Ltd Lanzhou 730000 China ji399211144@163.com

## Abstract

The escalating discharge of highly toxic cadmium (Cd(ii)) effluents presents critical threats to environmental security. To simultaneously address this remediation challenge and promote solid waste resource utilization, this study engineered a novel magnetic biochar (Fe@SBC) *via* the co-pyrolysis of excess municipal sludge and potassium ferrate (K_2_FeO_4_). Through orthogonal array optimization, specific synthesis parameters (pyrolysis at 600 °C for 2 h with a mass ratio of 2.0) were identified as critical for maximizing Cd(ii) remediation. Kinetic and isotherm modeling revealed that the adsorption process aligns with pseudo-second-order and Langmuir models, indicative of monolayer chemisorption. Under optimal conditions (pH 5.0, 25 °C), Fe@SBC attained a peak adsorption capacity of 155.28 mg g^−1^, nearly doubling the 83.89 mg g^−1^ capacity of unmodified sludge biochar. Thermodynamic analysis characterized the uptake as spontaneous, endothermic, and entropy-driven. Mechanistically, the superior performance of Fe@SBC was attributed to a synergy of surface complexation with oxygenated groups, inorganic precipitation, electrostatic attraction, and cation–π interactions. Furthermore, the material exhibited robust performance over five regeneration cycles. Given its cost-effectiveness, high efficiency, and magnetic separability, Fe@SBC is a promising candidate for sustainable heavy metal wastewater treatment.

## Abstract 2

The escalating discharge of highly toxic cadmium (Cd(ii)) effluents presents critical threats to environmental security.

## Graphical abstract

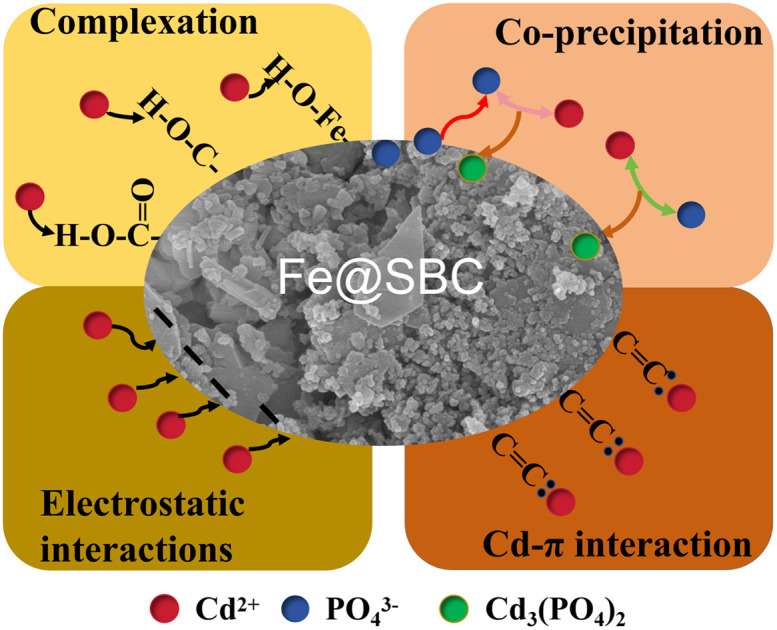

## 1. Introduction

The escalating expansion of sectors such as electroplating, mining, and battery production has precipitated a critical environmental challenge marked by the extensive release of heavy metal cadmium [Cd(ii)].^1,2^ Recognized by the World Health Organization (WHO) as a potent toxin, cadmium poses severe biological risks; its accumulation in the human body is linked to debilitating conditions, including skeletal degradation and renal dysfunction.^3^ Given these perils, there is an imperative need to engineer wastewater treatment strategies that are not only highly efficient but also economically sustainable to effectively curb these ecological and public health threats.

A spectrum of remediation techniques, ranging from membrane filtration and electrokinetic separation to adsorption, has been deployed to sequester Cd(ii) from aqueous environments.^4,5^ However, these conventional approaches are often constrained by significant practical limitations; for instance, membrane filtration is plagued by high operational costs and severe membrane fouling. Similarly, while emerging integrated technologies like electrokinetic-permeable reactive barriers demonstrate potential for *in situ* heavy metal remediation, their large-scale application is still severely hindered by substantial electrical energy consumption and complex field optimization.^6^ In contrast, adsorption is frequently prioritized owing to its exceptional cost-effectiveness, high scalability, and broad applicability across varied effluent concentration gradients. Particularly when utilizing waste-derived materials like biochar, adsorption not only provides an economically viable solution but also achieves the synergistic goal of solid waste resource utilization. While biochar—a porous, carbon-rich product of biomass pyrolysis—is a promising candidate,^7,8^ the application of pristine biochar is often constrained by its limited surface active sites and inherent negative surface charge, which curb its efficacy against Cd(ii).^9,10^ To circumvent these bottlenecks, surface engineering *via* metal oxide doping, particularly with iron (Fe), has emerged as a potent enhancement strategy.^2,11^ Recent advances have demonstrated that integrating iron species (*e.g.*, zero-valent iron or iron oxides) significantly enriches oxygen-containing functional moieties and magnetic separability, facilitating heavy metal capture through a synergy of complexation, electrostatic attraction, and precipitation mechanisms.^12–14^ Contemporary environmental management studies underscore that advanced Fe-integrated composites not only exhibit highly selective Cd(ii) sequestration but also offer superior environmental compatibility and structural stability compared to conventional materials.^1,9,15^ For instance, Liu *et al.*^16^ utilized a co-precipitation method to construct a porous confined nano-iron biochar nanoreactor, demonstrating that the spatial confinement effect significantly enhances the adsorption kinetics and capacities for heavy metals such as Pb and Cd. Furthermore, advanced mechanistic studies, such as the work by Mo *et al.*,^17^ have revealed that iron-based active sites (Fe–O) on magnetic biochar play a dominant role in the sequestration of divalent heavy metals through strong chemical coordination and interfacial charge transfer. Building upon these state-of-the-art advances, Yang *et al.*^18^ attributed the improved Cd(ii) uptake in Fe-modified biochar to carbonate precipitation and coordination with oxygenated groups. However, conventional iron modification protocols typically require cumbersome, multi-step processes involving sequential acid/base etching and secondary iron salt impregnation. In this context, potassium ferrate (K_2_FeO_4_) distinguishes itself as a green, dual-function agent: it acts simultaneously as a strong oxidant to etch the biochar surface and as an iron precursor to embed active sites during a single pyrolysis step. This one-pot simplicity eliminates the need for complex, time-consuming reagent mixtures, presenting a decisive advantage for large-scale, cost-effective adsorbent manufacturing.^19^

Despite these theoretical and practical advantages, systematic investigations into the co-pyrolysis of sludge biochar with K_2_FeO_4_ remain scarce, representing a critical knowledge gap in current environmental materials research. Consequently, the specific adsorption behaviors and the precise physicochemical mechanisms governing Cd(ii) removal by this composite material warrant in-depth exploration. In this work, we successfully synthesized a potassium ferrate-modified sludge biochar (Fe@SBC) *via* a one-step co-pyrolysis route. The study scrutinized the sensitivity of Cd(ii) removal to key environmental constraints, such as solution acidity, reaction time, and dosage levels. To decipher the adsorption process, we integrated mathematical modeling of kinetics and thermodynamics with microscopic structural analysis. The results obtained herein not only clarify the removal pathways but also underscore the potential of Fe@SBC as a scalable solution for the remediation of cadmium-laden wastewater.

## 2. Materials and methods

### 2.1 Reagents and material

Cadmium nitrate tetrahydrate [Cd(NO_3_)_2_·4H_2_O], nitric acid (HNO_3_), sodium hydroxide (NaOH), and potassium ferrate (K_2_FeO_4_) were of analytical grade and purchased from Sinopharm Chemical Reagent Co., Ltd (Shanghai, China). Cd(NO_3_)_2_·4H_2_O was accurately weighed and dissolved in deionized water (DW) to prepare Cd(ii) stock solutions of desired concentrations.

Pretreatment of raw materials: excess dewatered sludge was collected from a wastewater treatment plant in Chengdu. The sludge was first air-dried in a ventilated area for 7 days, followed by crushing into small particles. The particles were transferred to an oven and dried at 80 °C to a constant weight. Subsequently, the dried sludge was pulverized into powder and stored for further use.

### 2.2 Preparation of co-pyrolyzed modified biochar

A 1.0 g sample of sludge powder was weighed and thoroughly mixed with 0.5, 1.0, and 2.0 g of K_2_FeO_4_, respectively. The mixtures were filtered, dried, and then placed into a tubular furnace. Pyrolysis was conducted under an N_2_ atmosphere at a heating rate of 20 °C min^−1^. The samples were treated at pyrolysis temperatures of 400, 600, and 800 °C for durations of 0.5, 1.0, and 2.0 h, respectively, to prepare K_2_FeO_4_ co-pyrolyzed modified sludge biochar (Fe@SBC) under different conditions. The resulting samples were washed repeatedly with deionized water until the pH reached neutrality, dried at 80 °C to a constant weight, cooled, ground, and sealed for subsequent adsorption experiments. To evaluate the modification effect, unmodified sludge biochar (SBC) prepared without K_2_FeO_4_ addition under 600 °C and duration was used as a control.

### 2.3 Batch adsorption experiments

A 25 mL aliquot of 50 mg L^−1^ Cd(ii) and 20 mg of the adsorbent were added to polypropylene centrifuge tube. The solution pH was adjusted through the addition of 0.1 mol L^−1^ HNO_3_ or NaOH. The mixture was placed in a rotary shaker and agitated at 150 rpm and 25 °C for 600 min. The effects of adsorbent dosage (5–60 mg), initial solution pH (2.0–8.0), and coexisting ions (K^+^, Na^+^, Ca^2+^, Mg^2+^, Pb^2+^, Cu^2+^ and Zn^2+^) on removing Cd(ii) were investigated. Upon completion of the reaction, the mixture was passed through a 0.45 µm nylon membrane, and the remaining Cd(ii) concentration in the filtrate was quantified *via* atomic absorption spectrophotometry (AAS-6300, Shimadzu, Japan). All experiments were conducted in triplicate, and mean values were employed to determine the standard deviation. The sorption rate (*η*, %) and sorption amount (*q*_e_, mg g^−1^) were computed according to eqn (1) and (2), respectively.1
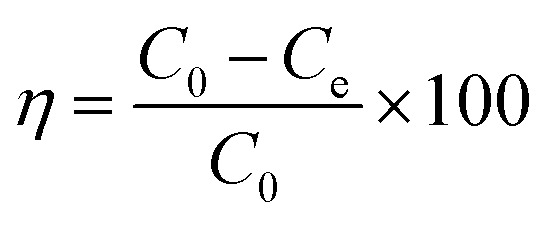
2
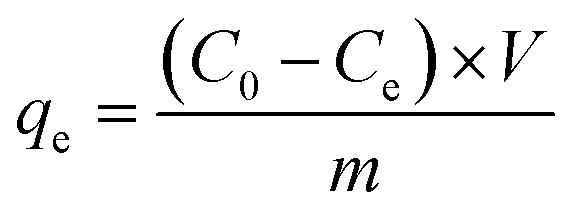
where *η* is the sorption rate (%); *C*_0_ and *C*_e_ are the initial and final concentration of Cd(ii) (mg L^−1^), respectively; *q*_e_ is sorption amount (mg g^−1^); *V* is the solution volume (mL); and *m* is the mass of adsorbent (mg).

#### 2.3.1 Adsorption kinetics

A series of centrifuge tubes were prepared corresponding to predetermined time intervals ranging from 1 to 600 min. In each tube, 50 mL of a 50 mg L^−1^ Cd(ii) solution and 20 mg adsorbent were added. The solution pH was regulated to 5.0 by adding 0.1 mol L^−1^ HNO_3_ or NaOH. Subsequently, the mixtures were transferred to a thermostatic shaker operating at 150 rpm and 25 °C for a reaction period of 600 min. The experimental data were interpreted using the pseudo-first-order, pseudo-second-order, Elovich and the intra-particle diffusion model (SI).

#### 2.3.2 Adsorption isotherms

A 50 mL volume of Cd(ii) solutions with varying initial concentrations (10–100 mg L^−1^) and 20 mg of the adsorbent were added to 50 mL centrifuge tubes. The solution pH was adjusted to 5.0 by adding 0.1 mol L^−1^ HNO_3_ or NaOH. Subsequently, the mixtures were transferred to a thermostatic shaker and agitated at 150 rpm and 25 °C for 300 min. The adsorption isotherm data were interpreted using the Langmuir, Freundlich and Temkin isotherm model (SI).

#### 2.3.3 Adsorption thermodynamics

To evaluate the thermal dependence of Cd(ii) uptake, batch equilibrium assays were executed across a controlled temperature gradient (15, 25, and 35 °C). Apart from temperature, all operational parameters were standardized: a solid-to-liquid ratio of 0.4 g L^−1^ (20 mg adsorbent dispersed in 50 mL of 50 mg L^−1^ Cd(ii) solution) was maintained at pH 5.0. The system was agitated at 150 rpm for a duration of 10 h (600 min) to ensure equilibrium. The resulting data facilitated the computation of key thermodynamic state functions-specifically standard Gibbs free energy (Δ*G*), enthalpy change (Δ*H*), and entropy change (Δ*S*)-as detailed in the SI.

#### 2.3.4 Reusability experiments

Reusability assessments commenced by saturating 20 mg of adsorbent with Cd(ii) (50 mg L^−1^, pH 5.0) under standard equilibrium conditions (25 °C, 150 rpm, 10 h). Following solid–liquid separation and quantification of the initial uptake, the Cd-laden residues were subjected to regeneration. This involved suspending the saturated biochar in a 0.1 M desorbing eluent and agitating it for 24 h at ambient temperature. The recovered solids were subsequently rinsed in triplicate with deionized water, thermally treated at 80 °C overnight, and re-employed in consecutive adsorption trials.

## 3. Results and discussion

### 3.1 Orthogonal experimental analysis

Instead of response surface methodology (RSM), the orthogonal experimental design (OED) was deliberately selected for this screening phase due to its high efficiency in determining the rank order of factor significance and its suitability for evaluating discrete thermal transformation levels with a minimal number of trials. An L9(3^3^) orthogonal array was constructed to optimize the synthesis protocol, specifically targeting the maximization of Cd(ii) adsorption rates. The experimental matrix scrutinized three pivotal variables: pyrolysis temperature (Factor A: spanning 400 to 800 °C), residence time (Factor B: 0.5 to 2 h), and the modifier-to-substrate mass ratio (Factor C: K_2_FeO_4_/sludge ratios of 0.5, 1, and 2). As shown in Table 1, The performance of each resultant variant was quantified based on its contaminant removal efficiency. Based on the experimental results from the nine groups, the *K* values (sum of removal rates), *k* values (average removal rates), and range (*R*) at different levels for each factor were calculated to determine the primary and secondary effects of each factor on response variable. The result indicated that the mean removal rates for pyrolysis temperature at the three levels were 81.49%, 92.15%, and 86.48%, respectively, with a corresponding range value *R*_A_ = 10.66. The average removal rates for pyrolysis time at the three levels were 83.45%, 87.59%, and 89.09%, respectively, yielding a range value *R*_B_ = 5.64. The average values for the K_2_FeO_4_/sludge mass ratio were 85.33%, 86.79%, and 87.67%, with a range value *R*_C_ = 2.34. The order of the range values for the factors was A > B > C, indicating that pyrolysis temperature was the dominant factor affecting Cd(ii) removal performance, followed by pyrolysis time. The influence of the K_2_FeO_4_/sludge ratio was relatively weak but still showed a trend of slight improvement with increasing ratio. Comprehensive analysis of the *k* values and range analysis suggested that the optimal combination of preparation levels was A2B3C3 (600 °C, 2 h, K_2_FeO_4_/sludge ratio of 2).

**Table 1:** Orthogonal experimental analysis of Cd(ii) removal by the adsorbent under different factors

Number	Factor 1	Factor 2	Factor 3
	Pyrolysis temperature (°C)	Pyrolysis time (h)	K_2_FeO_4_/sludge mass ratio (w/w)
1	400	0.5	0.5
2	400	1.0	1.0
3	400	2.0	2.0
4	600	0.5	1.0
5	600	1.0	2.0
6	600	2.0	0.5
7	800	0.5	2.0
8	800	1.0	0.5
*K*_1_	244.48	250.34	255.99
*K*_2_	276.45	262.77	260.37
*K*_3_	259.44	267.26	263.02
*k*_1_	81.49	83.45	85.33
*k*_2_	92.15	87.59	86.79
*k*_3_	86.48	89.09	87.67
*R*	10.66	5.64	2.34

### 3.2 Effect of environmental conditions

**Fig. 1:**
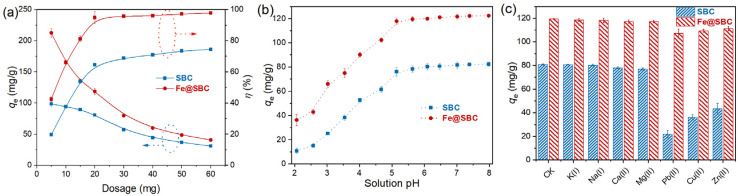
Effect of dosage (a), solution pH (b), and coexisting ions (c) on removing Cd(ii).

The dosage is a critical factor influencing the removal capacities of contaminants removal. As depicted in Fig. 1a, considering both adsorption efficiency and capacity, the most effective adsorbent dosage was identified as 20 mg. At a dosage of 20 mg, the sorption rate of Cd(ii) by SBC and Fe@SBC was 65% and 95%, respectively, with corresponding removal amount of 81 mg g^−1^ and 119 mg g^−1^. With a further increase in dosage, the removal rates increased slowly, while the adsorption capacities dropped sharply.^10^ Therefore, taking into account both removal rate and economic benefits, 20 mg was adopted as the optimal adsorbent dosage.

The solution pH was adjusted to a range of 2–8 to investigate effect on removing Cd(ii). As indicated in Fig. 1b, the adsorption performances of Cd(ii) improved as the pH increased from 2 to 5. This enhancement may result from the interaction of biochar functional group (*e.g.*, –COOH and –OH) with Cd(ii), leading to the formation of surface complexes.^10,20^ On the other hand, as the pH rose, the adsorbent surface transitioned from positively to negatively charged, facilitating electrostatic interactions arising from the opposite charges of the adsorbent and Cd(ii).^21^ Notably, in the pH range of 5–8, the removal capacities of SBC and Fe@SBC for Cd(ii) remained essentially constant. However, at higher pH levels (pH > 7), Cd(ii) may precipitate.^4^ Accordingly, pH 5 was determined to be the ideal pH for Cd(ii) uptake and was used in the ensuing experiments.

In actual wastewater, a large number of cations (*e.g.*, K^+^, Na^+^, Ca^2+^, Mg^2+^, Pb^2+^, Cu^2+^, and Zn^2+^) are often present. Thus, it is necessary to investigate the influences of cation on removing Cd(ii). As shown in Fig. 1c, K^+^, Na^+^, Ca^2+^, Mg^2+^ had almost no effects on removing Cd(ii) by SBC and Fe@SBC, with adsorption capacities maintaining around 118 mg g^−1^. However, when Pb^2+^, Cu^2+^, and Zn^2+^ were present as coexisting ions, the adsorption capacity for Cd(ii) decreased to 107, 109, and 111 mg g^−1^, respectively, indicating an antagonistic effect of these ions on Cd(ii) removal. The experimental results showed that the inhibitory ability followed the order: Pb^2+^ > Cu^2+^> Zn^2+^, which was attributed to the competitive adsorption behavior between these three heavy metals and Cd(ii) during the process.^10^ Furthermore, this inhibition may be due to the hydrolysis constants of Pb^2+^ (7.7), Zn^2+^ (9.0), and Cu^2+^ (8.0) being higher than that of Cd^2+^ (10.1).^4^ Therefore, the coexistence of Pb^2+^, Zn^2+^, and Cu^2+^ inhibited the Cd(ii) removal from the solution.

### 3.3 Adsorption behavior analysis

#### 3.3.1 Adsorption kinetics

**Fig. 2:**
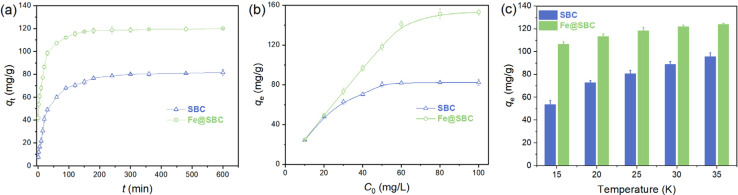
Effect of reaction time (a), concentrations (b), and temperatures (c) on removing Cd(ii).

To elucidate the influence of contact time on Cd(ii) removal, the removal performances of SBC and Fe@SBC were evaluated within 1–600 min. The adsorption capacity (Fig. 2a) of both materials increased progressively with reaction time. Overall, the adsorption proceeded through three distinct stages, including a fast uptake stage (1–30 min), a gradual adsorption stage (30–180 min), and an equilibrium stage (180–600 min). At the initial stage, abundant active adsorption sites and a large concentration gradient between the solid–liquid phases facilitated the rapid migration of Cd(ii) to the adsorbent surface and its immediate binding to the available sites, thus resulting in a sharp increase in adsorption capacity and rate.^19,22^ As the reaction progressed, the progressive occupation of surface-active sites diminished the solute concentration gradient between the solid and liquid phases, thereby weakening the primary driving force for mass transfer.^4,23^ This reduction triggered a mechanistic shift where adsorption became increasingly governed by intra-particle diffusion rather than film diffusion. Consequently, the uptake velocity decelerated significantly, tapering off until the system achieved equilibrium and the reaction rate effectively nullified.^10^

The kinetic parameters tabulated in Table 2 highlight the superior applicability of the pseudo-second-order (Fig. S1a) model (*R*^2^ = 0.999) over its pseudo-first-order (Fig. S1b) and Elovich (Fig. S1c) counterparts. This statistical alignment strongly advocates for a chemisorption-dominant pathway, likely orchestrated by electron sharing or exchange between Cd(ii) ions and surface functional moieties.^24,25^ Further dissection of the rate-controlling steps *via* the intra-particle model (Table S1 and Fig. S1d) revealed a characteristic tri-phasic profile. The process initiated with rapid external film diffusion driven by a steep concentration gradient,^10^ transitions into a slower intra-particle diffusion phase as Cd(ii) navigates the internal pore network,^9^ and concludes with a plateau representing the equilibrium state, where diffusion resistance peaks.^7,10^ According to Table S1, the sorption rates calculated for three stages followed the order: film diffusion > intra-particle diffusion > equilibrium stage. The deviation of the intra-particle diffusion plots from the origin indicated that intra-particle diffusion was not the only rate-limiting mechanism.^10^ Rather, the overall sorption processes were probably controlled by several mechanisms, such as film diffusion and ion–exchange reactions.

**Table 2:** Fitted sorption kinetic parameter for Cd(ii) removal by adsorbent

		SBC	Fe@SBC
	*q*_e,exp_	81.550	120.104
Pseudo-first-order	*q*_e,cal_	53.395	35.569
	*k*_1_	0.107 × 10^−1^	0.108 × 10^−1^
	*R* ^2^	0.961	0.848
Pseudo-second-order	*q*_e,cal_	84.818	121.212
	*k*_1_	0.053 × 10^−2^	0.139 × 10^−2^
	*R* ^2^	0.999	0.999
Elovich	*α*	13.921	363.801
	*β*	0.073	0.074
	*R* ^2^	0.966	0.948

#### 3.3.2 Adsorption isotherm

As shown in Fig. 2b, when Cd(ii) concentrations increased from 10 to 60 mg L^−1^, the uptake capacities of SBC and Fe@SBC rose sharply, reaching 81 mg g^−1^ and 141 mg g^−1^, respectively. As Cd(ii) concentration further rose to 100 mg L^−1^, the removal capacities of SBC and Fe@SBC rose more slowly, reaching 82 mg g^−1^ and 153 mg g^−1^. To gain deeper insight into the Cd(ii) sorption mechanisms of the biochars, the experimental results were interpreted using the Langmuir (Fig. S2a), Freundlich (Fig. S2b), and Temkin (Fig. S2c) models. As summarized in Table 3, the determination coefficient (*R*^2^) followed the order Langmuir > Freundlich and Temkin, demonstrating that the Langmuir model provided a superior representation of the sorption characteristics of SBC and Fe@SBC toward Cd(ii) in aqueous media.^7,19^ This finding implied that the sorption predominantly proceeded as a monolayer phenomenon on a structurally uniform surface.^5,8^ According to the Langmuir model, the estimated maximum removal amount of SBC and Fe@SBC for Cd(ii) at 25 °C were 84 mg g^−1^ and 155 mg g^−1^, respectively.

**Table 3:** Fitted adsorption isotherm parameter for Cd(ii) removal by adsorbent

		SBC	Fe@SBC
Langmuir	*q*_max_ (mg g^−1^)	83.893	155.280
	*K*_L_ (L mg^−1^)	1.076	0.587
	*R* ^2^	0.999	0.999
Freundlich	*K*_F_ (mg^1−*n*^ L^*n*^ g^−1^)	41.061	75.308
	*n*	4.969	3.655
	*R* ^2^	0.872	0.874
Temkin	*K*_T_ (g^−1^)	79.008	69.540
	*B*_T_ (kJ mol^−1^)	10.319	21.123
	*R* ^2^	0.948	0.924

To evaluate the Cd(ii) removal performance of the ferrate-modified sludge biochar synthesized in this work, its specific surface area and adsorption capacity (Table S2) were compared with those of various relevant biochar-based adsorbents reported in the literature.^16,26–30^ The synthesized material exhibited not only a high specific surface area of 117 m^2^ g^−1^ but also an exceptional Cd(ii) adsorption capacity of 155 mg g^−1^, demonstrating superior performance compared to all other evaluated adsorbents. In contrast, the adsorption capacities of the other materials varied significantly, ranging from 3 mg g^−1^ for porous ferrite-confined biochar to higher values of 149 mg g^−1^ for chitosan-modified magnetic biochar and 127 mg g^−1^ for S/Fe/Mn co-doped biochar.^16,26,29^ Furthermore, although the magnetic biochar-montmorillonite composite possessed a relatively low specific surface area of 21 m^2^ g^−1^, it achieved a substantial adsorption capacity of 125 mg g^−1^.^30^ This observation indicates that, alongside a well-developed porous structure, specific functional groups or chemically active sites introduced during the modification process play a crucial role in Cd(ii) adsorption. In summary, ferrate modification successfully endowed the sludge biochar with excellent textural properties and facilitated highly efficient Cd(ii) removal from aqueous solutions. These findings comprehensively demonstrate the substantial potential and practical advantages of this material for the remediation of heavy metal-contaminated water bodies.

#### 3.3.3 Adsorption thermodynamics

As illustrated in Fig. 2c, the Cd(ii) uptake amount of SBC and Fe@SBC exhibited an upward trend with the elevation of reaction temperatures from 15 to 35 °C. This enhancement could be ascribed to the increased availability of reactive site and the accelerated chemical reaction kinetics under elevated temperatures.^9,10^ The thermodynamic fitting parameters of Cd(ii) adsorption at different temperatures are summarized in Table 4 and Fig S3. The feasibility of the uptake process is quantitatively confirmed by the consistently negative values of Gibbs free energy (Δ*G*) for both adsorbents.^21,31^ Notably, the magnitude of −Δ*G* amplified with rising temperatures. This trend suggests that thermal energy inputs effectively overcome the activation energy barrier, thereby boosting the thermodynamic driving force and making Cd(ii) sequestration more favorable at higher temperatures.^7,20^ Concurrently, the positive enthalpy change (Δ*H*) categorizes the reaction as endothermic, necessitating heat absorption.^10^ Furthermore, the positive entropy value (Δ*S*) points to an increase in randomness at the solid–solution interface. This net gain in disorder likely stems from the displacement of hydration water molecules, which facilitates the affinity of Cd(ii) for the active sites on SBC and Fe@SBC.^21,32^

**Table 4:** Fitted thermodynamic parameters of adsorption

	Temperature (K)	Δ*G* (kJ mol^−1^)	Δ*H* (kJ mol^−1^)	Δ*S* (J mol^−1^ K^−1^)
SBC	288.15	−1.507	51.790	185.951
	293.15	−3.043		
	298.15	−3.762		
	303.15	−4.579		
	308.15	−5.364		
Fe@SBC	288.15	−6.400	113.370	413.866
	293.15	−7.778		
	298.15	−9.448		
	303.15	−11.574		
	308.15	−14.922		

### 3.4 Characterization and mechanisms

#### 3.4.1 Material characterization

**Fig. 3:**
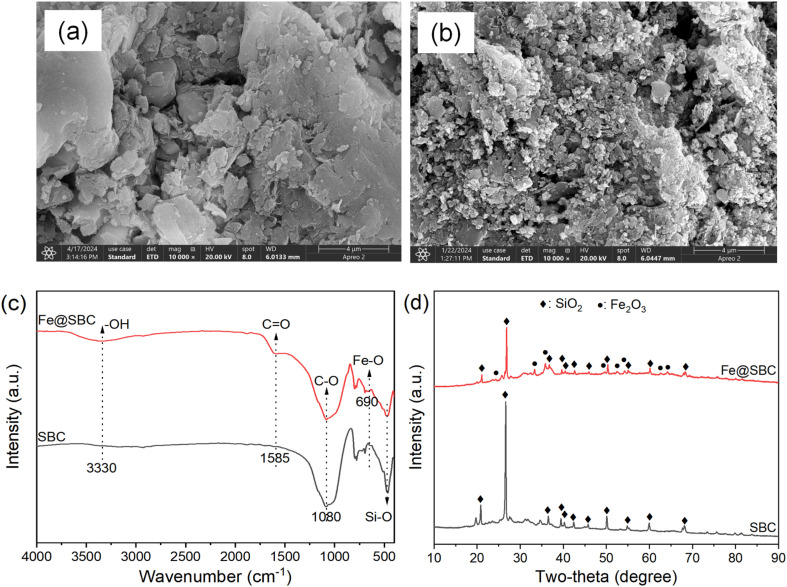
Morphological structure (a: SBC and b: Fe@SBC), FTIR (c), and XRD (d) analyses of biochar before and after modification.

As illustrated in the images, the unmodified pristine SBC (Fig. 3a) exhibits a relatively dense, smooth, and blocky surface morphology with limited pore development, which fundamentally restricts its available active sites for heavy metal adsorption. In stark contrast, following co-pyrolysis with potassium ferrate, the surface of the modified Fe@SBC (Fig. 3b) undergoes a dramatic transformation, becoming highly fragmented, distinctly rough, and porous. As presented in the Table 5, the unmodified SBC has a specific surface area of only 25.68 m^2^ g^−1^ and a pore volume of 0.089 cm^3^ g^−1^. However, upon co-pyrolysis with K_2_FeO_4_, the specific surface area of Fe@SBC surges to 117.24 m^2^ g^−1^ (an approximately 4.5-fold increase), accompanied by an expansion in pore volume to 0.229 cm^3^ g^−1^. This substantial improvement in the physical structure directly corroborates the potent oxidative etching effect of K_2_FeO_4_ during the high-temperature pyrolysis process, which effectively disrupts the original dense carbon skeleton of the sludge biochar to generate a highly developed internal pore network. Furthermore, the average pore diameter decreases from 16.51 nm in SBC to 10.19 nm in Fe@SBC. This reduction in average pore size is attributed to the generation of numerous new micropores and smaller mesopores during the severe etching process, which consequently lowers the overall average.

**Table 5:** Pore structure parameters of biochar before and after modification

	Specific surface area (m^2^ g^−1^)	Pore volume (cm^3^ g^−1^)	Average pore size (nm)
SBC	25.68	0.089	16.51
Fe@SBC	117.24	0.229	10.19

The structural and phase compositions of the materials before and after modification were analyzed using Fourier transform infrared spectroscopy (FTIR) and X-ray diffraction (XRD). As depicted in Fig. 3c, the unmodified SBC exhibits a relatively simple functional group profile, predominantly characterized by the C–O stretching vibration peak at 1080 cm^−1^ and the Si–O bending vibration peak near 460 cm^−1^ caused by inorganic ash.^4,22,25^ However, following modification, the spectrum of Fe@SBC not only retains the original characteristic skeleton peaks but also reveals the emergence of new reactive moieties: a broad –OH stretching vibration peak appears at 3330 cm^−1^, a newly formed C=O vibration peak at 1585 cm^−1^, and a distinct Fe–O bond signal is detected at 690 cm^−1^.^16,23,30^ These evolutions powerfully demonstrate that the modifier exerts a strong oxidative etching effect at high temperatures, disrupting the original carbon skeleton and introducing abundant oxygen-containing functional groups (–OH and C=O), while successfully loading iron species onto the biochar surface.^12,31^ Additionally, the XRD patterns (Fig. 3d) further reveal the crystalline phase transformations within the materials. The diffraction peaks of SBC are primarily assigned to silicon dioxide (SiO_2_), which is consistent with the inorganic characteristics of its sludge origin.^4,8,9^ In Fe@SBC, the dominant presence of SiO_2_ is preserved, indicating that the modification process does not destroy the rigid inorganic skeleton of the biochar, thereby ensuring the structural stability of the material. More crucially, new characteristic diffraction peaks definitively assigned to Fe_2_O_3_ emerge in the pattern.^17,26^ Corroborated by the detection of the Fe–O bond in FTIR analysis, this fully indicates that the iron-containing precursor underwent decomposition and crystallization during the pyrolysis process, ultimately anchoring firmly onto the biochar matrix in the form of crystalline Fe_2_O_3_ particles.

#### 3.4.2 Removal mechanism

**Fig. 4:**
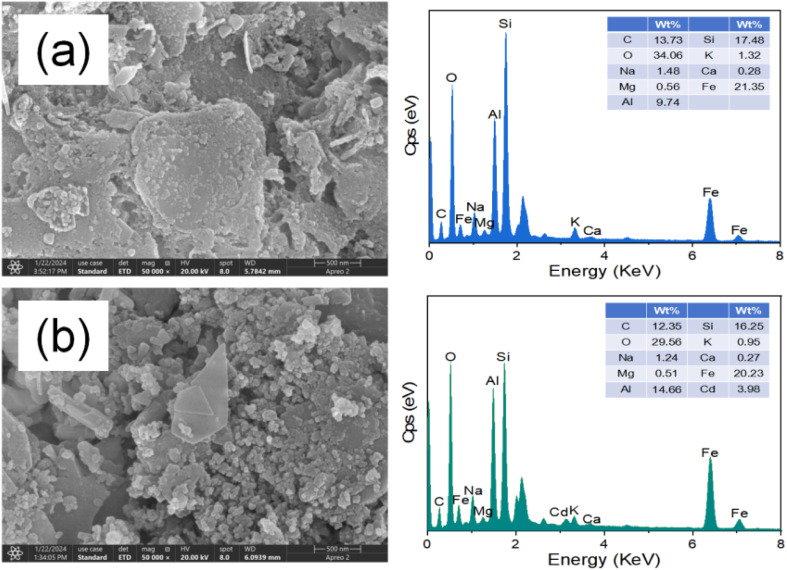
Morphology and elemental distribution of Fe@SBC before (a) and after (b) Cd(ii) removal.

**Fig. 5:**
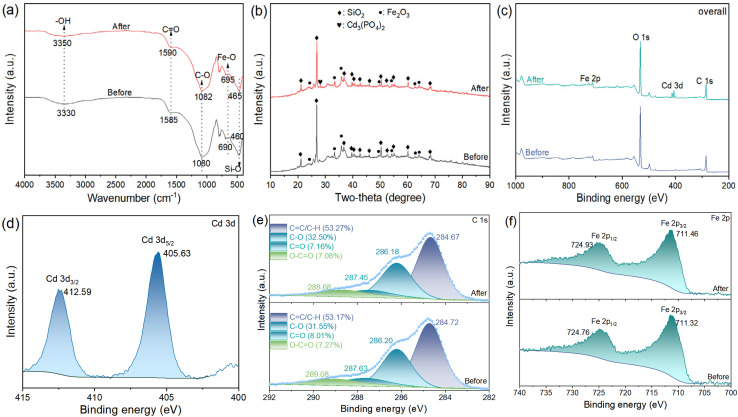
FTIR (a), XRD (b), XPS overall (c), Cd 3d (d), C 1s (e), and Fe 2p (f) spectra of Fe@SBC before and after Cd(ii) removal.

Morphological assessment *via* SEM (Fig. 4a and b) reveals that Fe@SBC preserves its heterogeneous, granular topography even after interacting with the contaminant, thereby attesting to its structural robustness. The successful capture of the target metal is further corroborated by EDS mapping, which detects emergent Cadmium signals on the post-adsorption surface. FTIR (Fig. 5a) provides molecular-level insights into the binding mechanism. Specifically, the blue shift of the –OH stretching vibration (3330 to 3350 cm^−1^), alongside the displacement of C=O (1585 to 1590 cm^−1^) and C–O bands (1080 to 1082 cm^−1^), indicates that surface oxygenated moieties—such as carboxyl and hydroxyl groups—act as primary coordination sites for Cd(ii).^19,33,34^ Furthermore, alterations in Fe–O (695 cm^−1^) and Si–O (465 cm^−1^) bands imply their participatory role in the sequestration process.^4,35^ In terms of crystallinity, XRD profiles (Fig. 5b) confirm that while the dominant SiO_2_ and Fe_2_O_3_ phases remain intact—reinforcing material stability—a new diffraction peak emerges near 2*θ* = 28.24°.^4,25,33^ This peak is ascribed to cadmium phosphate, providing tangible evidence that inorganic precipitation between released phosphates and free Cd(ii) ions constitutes a critical removal pathway.^4,10^

In the XPS survey spectrum of the spent adsorbent (Fig. 5c), distinct Cd 3d peaks emerged, further confirming the Cd(ii) removal. The high-resolution Cd 3d spectra (Fig. 5d) displayed two distinctive peaks assigned to Cd 3d_5/2_ and Cd 3d_3/2_ at binding energies of 405.63 eV and 412.59 eV, respectively.^34,36^ To clarify the contribution of O-bearing functional moieties to Cd(ii) sequestration, the C 1s spectra prior to and following sorption were examined in parallel (Fig. 5e). The C 1s spectrum of Fe@SBC consisted of four deconvoluted peaks: C=C/C–H (284.72 eV), C–O (286.20 eV), C=O (287.63 eV), and O–C=O (289.08 eV), with relative abundances of 53.17%, 31.55%, 8.01%, and 7.27%, respectively.^4,24^ After adsorption, all four binding energies shifted toward lower values (284.67, 286.18, 287.45, and 288.68 eV, respectively). Meanwhile, the relative content of C–O increased from 31.55% to 32.50%, while C=O and O–C=O decreased to 7.16% and 7.08%. These changes clearly demonstrated that oxygen-containing functional groups in Fe@SBC participated in Cd(ii) removal through complexation.^21,37^ Additionally, the decrease in the binding energy of C=C/C–H suggested that the carbon framework of Fe@SBC provided π electrons for interaction with Cd(ii), giving rise to cation–π interactions.^10^ In the Fe 2p spectra (Fig. 5f), Fe 2p_3/2_ and Fe 2p_1/2_ exhibited binding energies of 711.32 eV and 724.76 eV, respectively. Post-sorption, these peaks moved to 711.46 eV and 724.93 eV, suggesting that Fe–O group in Fe_2_O_3_ participated in Cd(ii) binding.^8,19^

**Fig. 6:**
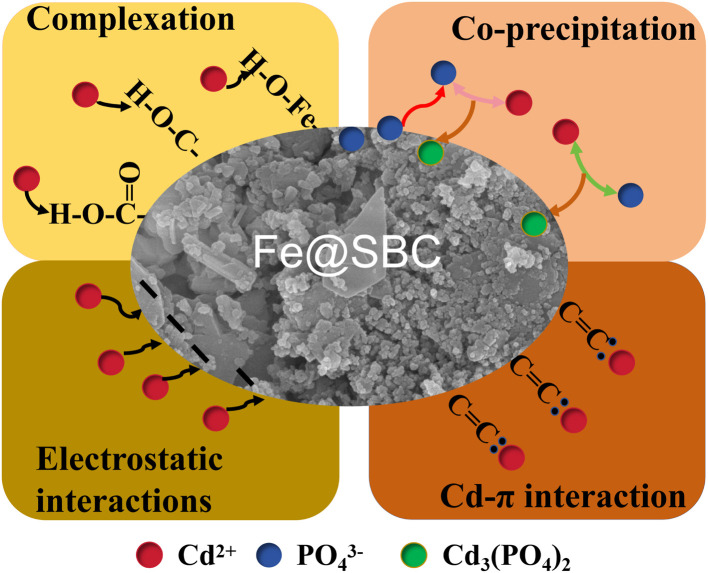
Schematic diagram of the potential mechanism for Cd(ii) removal by Fe@SBC.

In summary, the mechanisms (Fig. 6) governing the Cd(ii) removal by Fe@SBC include: (1) coordination interactions between Cd(ii) and oxygenated functional groups (*e.g.*, –OH, –COOH) present on the Fe@SBC surface; (2) cation–π interactions between Cd(ii) and the aromatic C=C domains of the biochar carbon skeleton; (3) electrostatic interactions between the negatively charged Fe@SBC and Cd^2+^; and (4) co-precipitation, wherein phosphate species released from Fe@SBC react with Cd(ii) to form cadmium phosphate precipitates.

### 3.5 Reusability and regeneration performance

**Fig. 7:**
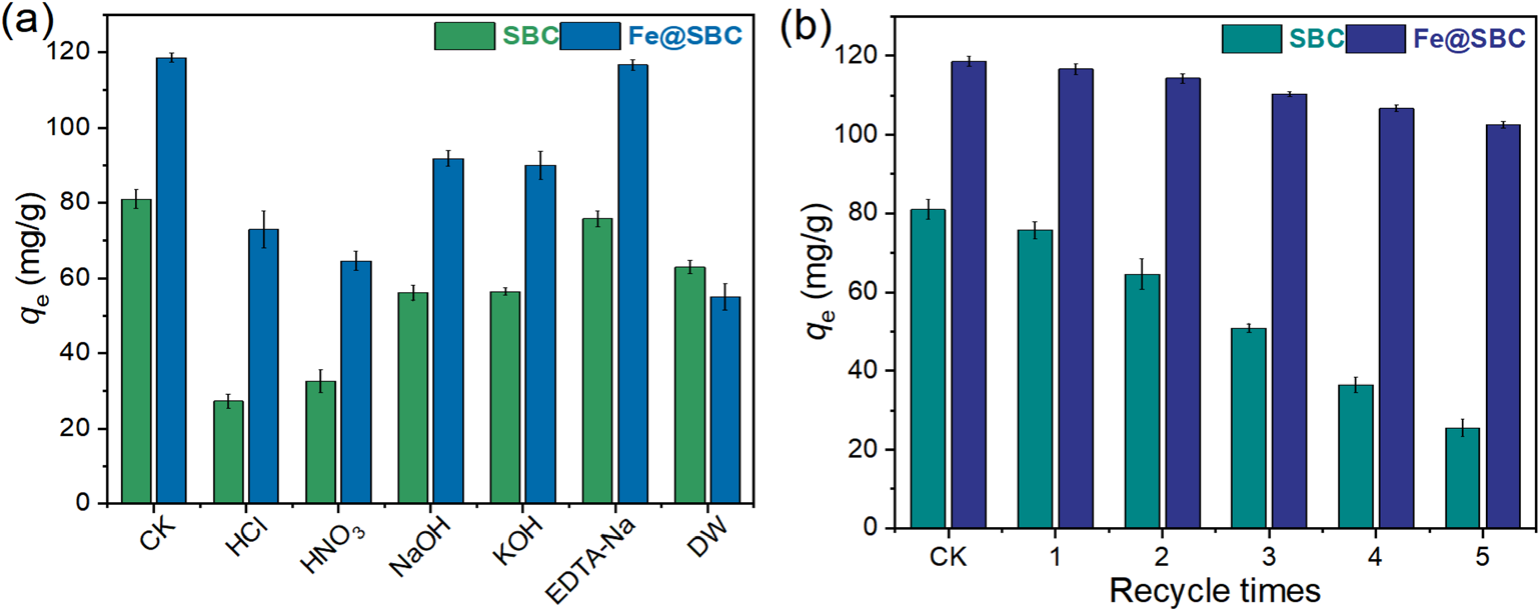
Removal performance under different regenerants (a) and after five regeneration cycles (b).

The regeneration ability of an adsorbent is a critical indicator for evaluating its economic feasibility and practical application potential. To identify the most effective desorption agent, six different 0.1 mol L^−1^ reagents (HCl, HNO_3_, NaOH, KOH, EDTA-Na, and DW) were first compared for their ability to regenerate SBC and Fe@SBC after Cd(ii) saturation. In Fig. 7a, the extent to which sorption amount was restored varied greatly among different regenerants. When DW was used, both SBC and Fe@SBC exhibited very limited recovery, indicating that Cd(ii) was strongly bound to the adsorbent surface and could not be removed by simple physical rinsing. Although acidic regenerants (HCl and HNO_3_) were capable of desorbing part of the heavy metals, the adsorption capacities declined substantially after regeneration (Fe@SBC decreased to approximately 65–75 mg g^−1^). Such diminished uptake under highly acidic conditions can be traced back to the structural degradation of pores or the saturation of binding sites with protons, both of which hinder Cd(ii) capture. In the comparative screening of regenerants, alkaline agents proved more effective than acids but fell short of the efficacy demonstrated by EDTA-Na. Among all tested reagents, EDTA-Na yielded the highest regeneration efficiency, achieving a recovery level of ∼117 mg g^−1^, which closely mirrors the initial capacity of 119 mg g^−1^. The rationale for this superior performance lies in EDTA's strong chelating potential; it facilitates the desorption of Cd(ii) *via* ligand exchange without compromising the material's physical stability. Given these advantages, 0.1 M EDTA-Na was designated as the optimal solvent for the cyclic adsorption–desorption experiments.

Utilizing the optimal regenerant, a five-cycle (Fig. 7b) durability protocol was executed to appraise the material's lifespan. While both adsorbents experienced some capacity attrition over successive runs, their degradation trajectories presented a stark contrast. The unmodified SBC suffered a precipitous decline, with its capacity shrinking from 81 mg g^−1^ to a mere 25 mg g^−1^. Conversely, the Fe@SBC nanocomposite demonstrated remarkable resilience, sustaining a capacity of 103 mg g^−1^ in the final cycle—a marginal drop from its initial 118 mg g^−1^. This minor performance decay is likely a consequence of the cumulative blockage of pores or the irreversible occupation of high-affinity sites by residual contaminants.^4,10^ Ultimately, with over 80% capacity retention, Fe@SBC proves that potassium ferrate co-pyrolysis not only boosts adsorption power but significantly fortifies the structural integrity, validating its potential for long-term industrial deployment.

## 4. Conclusions

In this work, a highly efficient magnetic biochar (Fe@SBC) was successfully engineered *via* the one-step co-pyrolysis of excess municipal sludge and K_2_FeO_4_, effectively demonstrating a sustainable “waste-to-wealth” strategy. Rather than merely reporting optimal parameters, our findings highlight the fundamental transformation of the sludge matrix, where K_2_FeO_4_ acted synergistically to etch the pore structure and homogeneously embed iron-oxygen active sites. This unique structural evolution drove a multifaceted Cd(ii) sequestration mechanism encompassing surface complexation, cation–π interactions, electrostatic attraction, and cadmium phosphate precipitation, resulting in an exceptional adsorption capacity. Beyond theoretical insights, Fe@SBC exhibited robust practical viability, characterized by its high resistance to common background alkali/alkaline earth cations and excellent structural stability over multiple regeneration cycles. Ultimately, this work not only provides a cost-effective and scalable adsorbent for heavy metal remediation but also proposes a viable pathway for the resource utilization of municipal sludge. Future investigations will focus on the pilot-scale application of Fe@SBC and its performance in treating complex, multi-contaminant industrial wastewater matrices.

## Author contributions

Tao Long: conceptualization; methodology; investigation; data curation; formal analysis; visualization; writing-original draft. Xinwei Zuo: methodology; validation; writing-review & editing. Yunping Ji: investigation; resources; project administration; visualization; writing-review & editing. Changquan Wang: conceptualization; supervision; writing-review & editing.

## Conflicts of interest

The authors declare that they have no known competing financial interests or personal relationships that could have appeared to influence the work reported in this paper.

## Supplementary Material

RA-016-D6RA00674D-s001

## Data Availability

Data will be made available on request. Supplementary information: detailed equations for the adsorption kinetics, isotherms, and thermodynamic models (Text S1), as well as descriptions of the material characterization technologies used, including SEM-EDS, XRD, and FTIR (Text S2). Additionally, it provides data tables detailing the intra-particle diffusion model fitting results (Table S1) and a literature comparison of maximum Cd(ii) adsorption capacities (Table S2). The file also includes supplementary figures showing the fitting curves for the kinetic, isotherm, and thermodynamic models (Fig. S1–S3), along with the associated references. See DOI: https://doi.org/10.1039/d6ra00674d.
