# Supplementary material for: Removal of Cd(ii) from aqueous solution by ferrate-modified sludge biochar: optimization of preparation conditions, adsorption performance, and mechanism
Source: RSC Adv. 2026 May 6;16(26):23730–9. doi: 10.1039/d6ra00674d (PMC13147236; doi:10.1039/d6ra00674d)
Supplement: RA-016-D6RA00674D-s001 [file RA-016-D6RA00674D-s001.pdf]

Supplementary Information (SI) for RSC Advances.
This journal is © The Royal Society of Chemistry 2026

Removal of Cd(II) from Aqueous Solution by Ferrate-Modified Sludge Biochar: Optimization of Preparation Conditions, Adsorption Performance, and Mechanism

Tao Long [a], *Xinwei Zuo* [b], *Yunping Ji* [c,*], *Changquan Wang* [a,*]

[a] *College of Resources, Sichuan Agricultural University, Chengdu 611130, China.*

[b] *China Nuclear Industry Survey Design & Research Co., Ltd., Zhengzhou 450002, China.*

[c] *China Railway First Survey and Design Institute Group Co., Ltd, Lanzhou 730000, China*

**Corresponding author: Yunping Ji (shaopei13698@163.com); Changquan Wang (*chang10393@163.com*)*

Text S1 Adsorption model

Adsorption kinetics model: the pseudo-first-order model (Eq. S1), the pseudo-second-order model (Eq. S2), Elovich model (Eq. S3), and intra-particle diffusion model (Eq. S4) were used for fitting analysis.

$$q_t = q_e(1-e^{-k_1 t}) \qquad \text{(S1)}$$

$$q_t = \frac{q_e^2 k_2 t}{1+q_e k_2 t} \qquad \text{(S2)}$$

$$q_t = \beta \ln(\alpha\beta) + \beta \ln(t) \qquad \text{(S3)}$$

$$q_t = K_{id} t^{1/2} + C_i \qquad \text{(S4)}$$

Where q_e and q_t are the sorption capacity at the equilibrium time and time "t" time (mg/g), respectively; k_1 and k_2 represent sorption rate constant of the pseudo-first-order (1/min) and the pseudo-second-order (g/mg·min), respectively; K_{id} (mg/g·min$^{1/2}$) is rate constants of intra-particle diffusion, respectively; C_i is constant of the intra-particle diffusion model.

Adsorption isotherm model: the Langmuir model (Eq. S5), Freundlich model (Eq. S6), and Temkin model (Eq. S7) were used to fit these data.

$$\frac{C_e}{q_e} = \frac{1}{q_{max} K_L} + \frac{C_e}{q_{max}} \qquad \text{(S5)}$$

$$\ln q_e = \ln K_F + \frac{\ln C_e}{n} \qquad \text{(S6)}$$

$$q_e = B_T \ln A_T + B_T \ln C_e \qquad \text{(S7)}$$

Where q_e is the sorption capacity at equilibrium (mg/g); C_e is the concentration of Cd(II) at sorption equilibrium (mg/L); q_{max}, and K_L are the maximum sorption capacity of Cd(II) (mg/g), and Langmuir equilibrium constant (L/mg), respectively; K_f and n represent the Freundlich affinity coefficient (mg^{1-n}·L^n/g) and Freundlich constant related to the surface site heterogeneity, respectively; A_T (1/g) and B_T (kJ/mol) are Temkin constants.

The thermodynamic investigation of Cd(II) removal at different temperatures (15~35°C). The thermodynamic parameters for the removal process were calculated using Eq. S8 and Eq. S9.

$$\ln K_d = \frac{\Delta S}{R} - \frac{\Delta H}{RT} \qquad \text{(S8)}$$

$$\Delta G = -RT\ln K \qquad \text{(S9)}$$

where, R is the gas constant (8.314 J/mol·K). K is the equilibrium constant and T is the Kelvin temperature (K). The ΔG, ΔH and ΔS represent the standard free energy (k·J/mol), enthalpy (k·J/mol), and entropy (J/mol·K), respectively.

Text S2 Characterization technologies

The surface microstructure of the material was observed using a scanning electron microscope equipped with an energy-dispersive spectrometer (SEM-EDS, JSM-6490LV, JEOL, Japan). The phase composition of the material was characterized using an X-ray diffractometer (XRD, Ultima IV, Rigaku, Japan). The surface functional groups of the material were determined using a Fourier-transform infrared spectrometer (FTIR, Nicolet 6700, Thermo Fisher Scientific, USA).

Table S1 Intra-particle diffusion model fitting results for Cd(II) removal.

Model	Parameter	SBC	Fe@SBC
Intra-particle diffusion	C_1	-1.938	33.504
	K_{d1} (mg/g·min$^{1/2}$)	9.033	11.602
	R_1^2	0.980	0.984
	C_2	29.582	98.548
	K_{d2} (mg/g·min$^{1/2}$)	3.506	1.515
	R_2^2	0.990	0.984
	C_3	76.620	116.349
	K_{d3} (mg/g·min$^{1/2}$)	0.198	0.144
	R_3^2	0.898	0.938

Table S2 Comparison of the maximum adsorption capacity of Cd (II) by different types of adsorbents.

Adsorbent	specific surface area (m^2/g)	Maximum adsorption capacity (mg/g)	References
Ferrite porous confined biochar	77.39	2.46	[1]
Biochar-based magnetic nanocomposite	/	38.60	[2]
$KMnO_4$ modified biochar	/	122.10	[3]
Magnetic biochar-montmorillonite composites	20.60	124.71	[4]
Biochar co-doped with S, Fe, and Mn	/	127.37	[5]
Chitosan-Modified Magnetic Biochar	112.08	149.30	[6]
Ferrate-Modified Sludge Biochar	117.24	155.28	This work

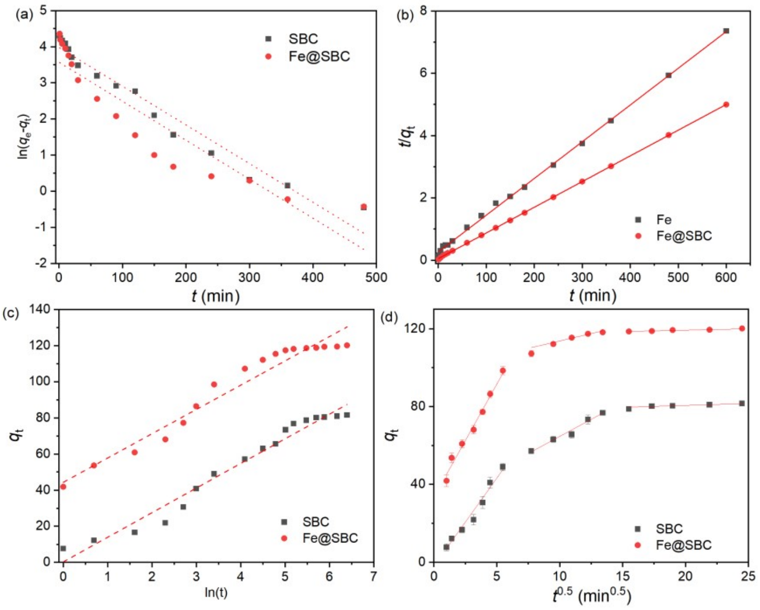

Fig. S1 Fitting curves of the pseudo-first-order (a), pseudo-second-order (b), Elovich (c), and intra-particle (d) models for Cd(II) removal by SBC and Fe@SBC.

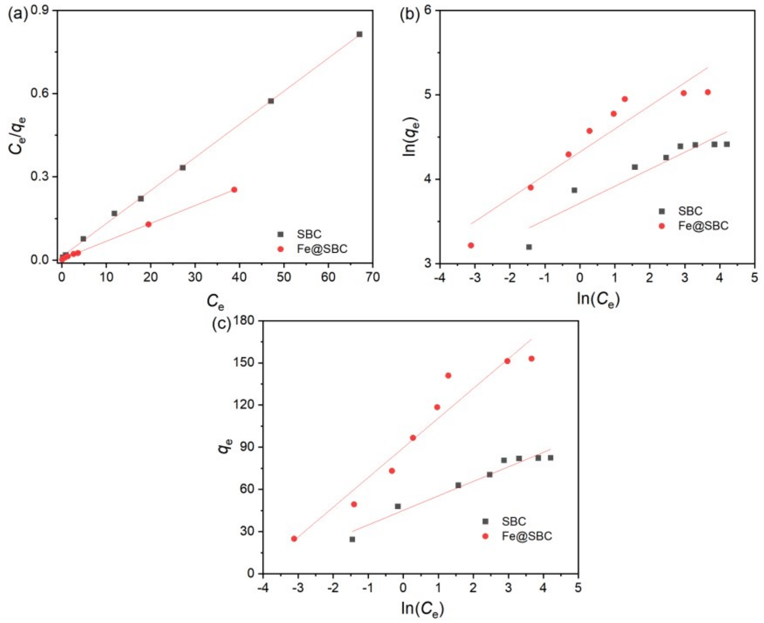

Fig. S2 Fitting curves of the Langmuir (a), Freundlich (b), and Temkin (c) models for Cd(II) removal by SBC and Fe@SBC.

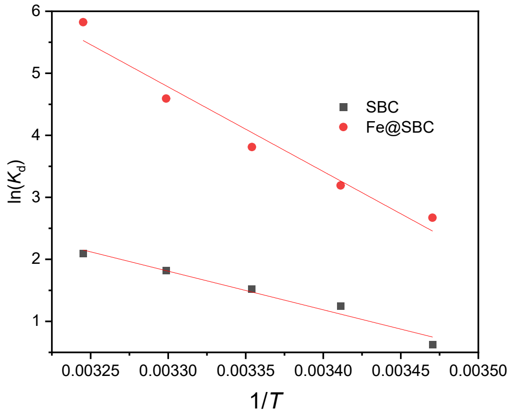

Fig. S3 Thermodynamic fitting curve.

1. N. Liu, R. X. Zhou, S. X. Huang, J. M. Zhang, H. D. Zhang, H. Liu, X. Du, D. Y. Zhang, R. L. Zhang, Z. T. Sun, S. F. Fu and X. M. Zhan, *J. Water Process. Eng.*, 2025, **74**, 107717.
2. N. Zhang, F. Reguyal, S. Praneeth and A. K. Sarmah, *Environ. Pollut.*, 2023, **330**, 121806.
3. X. R. Liang, X. F. Rao, Q. L. Fu, J. Zhu and H. Q. Hu, *J. Environ. Chem. Eng.*, 2023, **11**, 109874.
4. Y. Zhao, J. Yao, N. Min, B. Ma, W. P. Chai and Y. Q. Ma, *Separation and Purification Technology*, 2026, **391**, 136996.
5. H. Zhang, H. Y. Huang, Y. R. Fang, J. M. Cai, X. Y. Lv, W. L. Wu, Q. Gu, R. Qian, K. R. Yu, K. H. D. Tang and R. H. Li, *Separation and Purification Technology*, 2025, **363**, 132025.
6. R. Kareem, A. Afkhami and K. H. H. Aziz, *ChemistrySelect*, 2025, **10**, e03708.
